# Identification of variables contributing to superovulation efficiency for production of transgenic prairie voles (*Microtus ochrogaster*)

**DOI:** 10.1186/1477-7827-10-54

**Published:** 2012-07-27

**Authors:** Alaine C Keebaugh, Meera E Modi, Catherine E Barrett, Chengliu Jin, Larry J Young

**Affiliations:** 1 Center for Translational Social Neuroscience, Department of Psychiatry and Behavioral Neuroscience, Emory University School of Medicine, Division of Behavioral Neuroscience and Psychiatric Disorders, Yerkes National Primate Research Center, Atlanta, GA, USA; 2 Transgenic and Gene Targeting Core, Georgia State University, Atlanta, GA, USA

**Keywords:** Prairie vole, Superovulation, Social behavior, Transgenic, Animal model

## Abstract

**Background:**

The prairie vole (*Microtus ochrogaster*) is an emerging animal model for biomedical research because of its rich sociobehavioral repertoire. Recently, lentiviral transgenic technology has been used to introduce the gene encoding the green fluorescent protein (GFP) into the prairie vole germline. However, the efficiency of transgenesis in this species is limited by the inability to reliably produce large numbers of fertilized embryos. Here we examined several factors that may contribute to variability in superovulation success including, age and parentage of the female, and latency to mating after being placed with the male.

**Methods:**

Females produced from 5 genetically distinct breeder lines were treated with 100 IU of pregnant mare serum gonadotrophin (PMSG) and immediately housed with a male separated by a perforated Plexiglas divider. Ovulation was induced 72 hr later with 30 IU of human chorionic gonadotropin (hCG) and 2 hrs later mating was allowed.

**Results:**

Superovulation was most efficient in young females. For example, females aged 6-11 weeks produced more embryos (14 +/- 1.4 embryos) as compared to females aged 12-20 weeks (4 +/- 1.6 embryos). Females aged 4-5 weeks did not produce embryos. Further, females that mated within 15 min of male exposure produced significantly more embryos than those that did not. Interestingly, there was a significant effect of parentage. For example, 12 out of 12 females from one breeder pair superovulated (defined as producing 5 or more embryos), while only 2 out of 10 females for other lines superovulated.

**Conclusions:**

The results of this work suggest that age and genetic background of the female are the most important factors contributing to superovulation success and that latency to mating is a good predictor of the number of embryos to be recovered. Surprisingly we found that cohabitation with the male prior to mating is not necessary for the recovery of embryos but is necessary to recover oocytes. This information will dramatically reduce the number of females required to generate embryos for transgenesis in this species.

## Background

The socially monogamous prairie vole (*Microtus ochragaster*) is an excellent model organism for understanding the genetics and neurobiology regulating social bonding and other behaviors associated with monogamy [1], which are not exhibited by polygamous laboratory mouse and rat species. Because prairie voles can be systematically outbred they are ideal for the study of individual variation in neurochemistry and sociobehavioral traits [2,3]. Over two decades of research in this species has provided insights into the neurobiological basis of social attachment [4,5] and nurturing behavior [3,6-8], and voles have served as a model of how social experience effects adult social behavior [9], depression [10,11] and cardiac function [12]. Discoveries in prairie voles are beginning to inform novel treatment strategies for psychiatric disorders with impairments in social behavior [13,14]. The prairie vole is of great interest for biomedical research and the ability to genetically manipulate this non-traditional animal model would allow for the study of diseases associated with social deficits in a more behaviorally relevant species.

There is an ongoing effort within the prairie vole research community to develop comprehensive genomic resources to facilitate biomedical research in this model organism, including a 10X BAC library [15,16], a cytogenetic and genetic linkage map [17], and the genome is forthcoming. Recently, lentiviral mediated transgenic technology was used to introduce the green fluorescent protein (GFP) gene into the prairie vole germline, as a proof of principle [18]. Progress is being made combining this approach with RNAi technology to silence gene expression, but the inefficiency of superovulation and embryo transfer has been a significant impediment to further use of this technology to explore genetic mechanisms of behavior. Further, the powerful technology of gene targeting using homologous recombination has not yet been applied to the prairie vole. The future success of these transgenic technologies in this species requires reliable methods of superovulation that yield large numbers of viable oocytes and embryos with normal developmental potential.

Superovulation is a procedure used to produce a large number of developmentally synchronized embryos and protocols based on administration of gonadotrophic hormones have been standardized in species such as mouse [19], rat [20,21], pig [22], cow [23], rabbit [24] and goat [25]. However, the responsiveness of each species to superovulation treatment varies and must be optimized to account for species differences [26,27]. Within the laboratory mouse, optimal age, hormone dose, and other factors vary between strains [28]. Further, among rats there is also considerable variation between laboratories with respect to the choice of strain, optimal age, hormone and hormone dose [29,30].

In addition to the prairie vole’s unusual social system (characterized by social monogamy, formation of extended families, and cooperative breeding) [31,32], their reproductive physiology differs significantly from traditional laboratory rodents [33,34]. Prairie voles are unusual in that they are more responsive to social factors rather than environmental cues to reach estrus [33-35]. Behavioral estrus in the prairie vole occurs 1-3 days after the female is introduced to a novel male or male urine and ovulation is typically induced following mating [33,36,37]. Prairie vole’s mate repeatedly for 24 h (3-31 bouts) [38] and successful reproduction in this species requires prolonged contact with a male [39,40]. The social cues initiating the ovarian development are olfactory [41,42]. Estrus synchronization, superovulation and fertilization represent a significant challenge for the efficient production of transgenic prairie voles. Since prairie voles are not currently commercially available, meaning that donor females are often limiting, it is essential to understand the variables that can contribute to successful embryo harvesting. In our own experience, inconsistency in the success of superovulation and fertilization has been a significant barrier to efficient transgenesis. Thus, the main goal of this study was to explore some of the variables that contribute to superovulation success in the prairie vole. We examine several factors that are known to contribute to variability in superovulatory success in other species including age of female, latency to mate following exposure to a male, and parental lineage of the female. Further, the single published study inducing superovulation in the prairie vole incorporates separated cohabitation with a male combined with hormone administration [18]; however, the need for this extra step (i.e. separated cohabitation) is just a hypothesis. Thus, we test the importance of including this extra step. We describe in detail the method for inducing synchronized ovulation via hormonal manipulation (pregnant mare serum gonadotrophin / human chorionic gonadotropin) *without* the need for sociosexual manipulation, a time intensive procedure that requires specialized housing.

## Methods

### Subjects

Subjects were sexually naive female prairie voles 4-20 weeks old, and stud males were adult (90–365 days of age), sexually experienced prairie voles. All prairie voles were generated from an in-house breeding colony originally derived from Illinois prairie voles. After weaning at 21 days of age, subjects were housed in same sex pairs or trios with water and Purina rabbit chow provided *ad libitum* under a 14:10 light:dark cycle with lights on at 7 am. All experiments were done in accordance to the Institutional Animal Care and Use Committee at Emory University.

### Superovulation protocol: separated cohabitation & hormone treatment

Prairie voles do not display spontaneous ovarian activity or ovulation. Exposure to olfactory scents from a male is necessary to induce sexual receptivity and follicle development under nonhormonally-primed conditions [34,36,42]. Once receptive, ovulation occurs only after 10 or more hours of pairing after mating takes place [38]. To induce receptivity and synchronize ovulation in multiple animals, each naive female was either housed in a cage with a sexually experienced stud male but separated from him by a Plexiglass divider or returned to her home cage. Prior to pairing, to increase the number of mature follicles, females were administered 100 IU of PMSG intraperitoneally [43] at 3 PM immediately before being placed into a separated cohabitation with the stud male. Seventy-two hours later females were administered 30 IU IP of hCG at 3 PM to induce ovulation [43]. Two hours later at 5 PM the divider was removed to allow mating. In experiment 1, control animals were injected with sterile saline on the same time schedule as PMSG and hCG as described above. As previously shown for the polygamous Japanese field vole, *Microtus montebelli*[43], Experiment 1 demonstrates that this hormone regimen is effective at inducing superovulation in the prairie vole in the absence of pre-exposure to a male (e.g. separated cohabitation). Much of this data, however, was collected retrospectively; thus, in experiments 2 and 3 the hormone and separated cohabitation was held constant in order to explore the contribution of other variables. It should be noted that this high hormone dose was chosen based on pilot studies testing various PMSG/hCG doses (5 IU/5 IU as is done in mice and rats, 25 IU/25 IU, 50 IU/25 IU and 75 IU/25 IU) which failed to reliably induce superovulation (Keebaugh and Young, unpublished data).

### Oocyte and embryo harvesting

Seventeen hours following administrations of hCG females were sacrificed using CO_2_ asphyxiation (approximately 8 AM), their oviducts removed and placed into M2 media (Millipore, Billerica, MA). Under a stereoscope, a 32 gauge needle was placed into the infundibulum and oviducts were flushed with ~0.3 ml M2 media. Harvested embryos were stored in M16 media (Millipore) microdrops under mineral oil at 37 °C and 5% CO_2_.

### Experiment 1. Effectiveness of hormone administration and pre-exposure to a male on oocyte and embryo production

Two studies were conducted to determine the importance of hormone administration and pre-exposure to a male on oocyte (no mating) and embryo (mating) production. For each experiment, females 9-10 weeks of age i) were given either saline/saline or PMSG/hCG and ii) underwent either separated cohabitation or were singly housed prior to saline or hCG treatment (n = 15/treatment). Superovulation and oocyte/embryo harvesting protocols were done as described above. Females producing no oocytes/embryos were considered not to have ovulated; females producing 1-6 oocytes/embryos were classified as having ovulated; females producing more than 7 oocytes/embryos were classified as having superovulated since the typical litter sizes are 3-5 pups.

In experiment 1a we examined oocyte production; thus, females did not mate. Treatment group 1.1 (G1.1) served as the control. They received saline, did not mate, and were singly housed. To determine if hormone administration and/or pre-exposure to a male would lead to differences in the number of oocytes produced, treatment group 1.2 (G 1.2) and treatment group 1.3 (G 1.3) females both received hormone but G1.2 females were singly housed while G1.3 females were pre-exposed to a male via separated cohabitation.

In experiment 1b we looked at embryo production; thus, females did mate. Females in treatment group 2.1 (G2.1) received saline and were pre-exposed to a male. Treatment group 2.2 (G2.2) and treatment group 2.3 (G2.3) females both received hormone but G2.2 females were singly housed while G2.3 females were pre-exposed to a male.

### Experiment 2. Importance of female age and occurrence of mating as indicators of superovulatory success

Females ranging in age from 4-20 weeks old were given PMSG/hCG according to the superovulation protocol described. Mating was scored as occurring or not occuring during the first 15 minutes after removing the divider and embryos were collected the following morning beginning at 8 AM.

### Experiment 3. Role of female parentage in superovulatory efficiency

Previous studies have identified variation in response to superovulation (i.e. high versus low responders) across substrains of mice. Among Illinois derived laboratory prairie vole colonies it has been reported that about half of females exposed to males for 2-3 days will fail to show lordosis [44]. Our prairie vole colony is derived from Illinois and maintained as an outbred population; thus we are interested in identifying if genetic background contributes to variation in superovulatory response and ultimately genetic lines optimal for inducing superovulation. Females 7-11 weeks of age from five breeder pairs (N = 36; BP1 = 7, BP2 = 7, BP3 = 7, BP4 = 9, BP5 = 6) were given PMSG/hCG according to the superovulation protocol described above.

### Statistical analysis

For experiment 1 the Freeman-Halton extension of Fisher’s exact test was used to compute two-tailed probabilities of obtaining a distribution of values in a 3x3 contingency table for each experimental group. For oocyte and embryo production, statistical analysis was used to determine 1) is hormone priming critical for superovulation and 2) is pre-exposure to a male important. For experiment 2 regression analysis was used to determine the predictive value of female age (n = 85) on the number of embryos recovered. A chi-square test was used to determine if the occurrence of mating within the first 15 minutes of male access (n = 85) contributed to superovulatory response. For experiment 3 one-way ANOVAs were run to compare the number of embryos produced between breeder pairs. Hochberg’s GT2 test was used for post hoc analysis when significant effects were detected.

### Experiment 4. Generation of transgenic prairie voles

Production of lentivirus*.* We used the pLenti DEST vector (Gateway cloning, Invitrogen) which contains a mU6 driven shRNA and a CMV driven GFP coding sequence. The shRNA was annealed and ligated into the pENTR/U6 kan vector using the oligos 5^′^-CACCGTGGATCACGCTTGCCGTCTACATTGCGAACAATGTAGACGGCAAGC GTGATCCA-3^′^ and 5^′^- aaaaTGGATCACGCTTGCCGTCTACATTGTTCGCAATGTAGACGGCAAGCGTGATCCAC-3^′^. The resulting pENTR/U6 kan + oligos vector was recombined using the gateway kit with the pLenti DEST vector.

**Figure 1:**
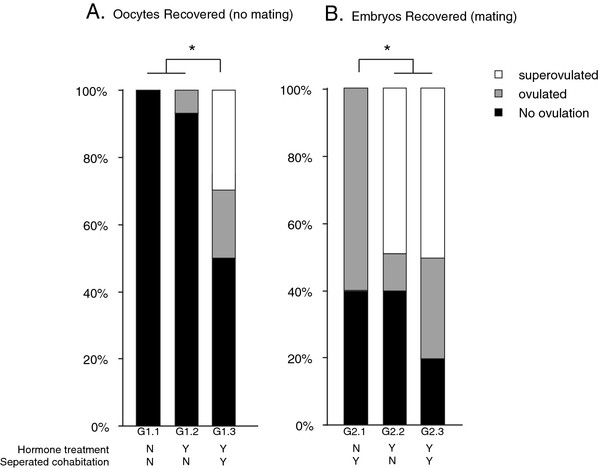
**Impact of hormone administration and separated cohabitation on superovulatory response in the presence and absence of mating.** The GFP expressing lentiviral vector harboring a shRNA targeting oxtr mRNA at nucleotide site 607 is shown in (**A**) and was injected into the perivetilline space of single-cell embryos harvested from pregnant female prairie voles. (**B**) Polymerase chain reaction analysis indicates the presence of the GFP transgene in a single founder animal.

To produce lentivirus, the viral vector was cotransfected with plasmid p(Δ)8.9 and pVSVG into HEK 293FT packaging cells. Supernatant was collected and concentrated by ultracentrifugation. The resulting concentration of infectious particles was determined by expression of GFP in HEK293FT cells plated at a density of 2.5 × 10^5^ per well in a six-well plate. Titer was determined by multiplying the number of GFP-positive cell colonies by the dilution factor and presented as colony-forming units (cfu/ml). We aliquoted high-titer virus (1 × 10^9^ cfu/ml; 5ul per tube) and stored it at -80 C until use.

Production of sterile stud males. Sexually experienced adult male prairie voles were vasectomized and used to induce pseudopregnancy. An incision was made at the caudal end of the abdominal cavity, and the vas deferens were located and cauterized. Males were allowed to recover for 2 weeks and then cohabitated with a female for 4 weeks to ensure sterility. Only confirmed sterile males were used to induce pseudopregnancy. Vasectomized males were used in multiple experiments and retired once they reached 1 year of age.

Production of Psuedopregnant recipient females. Surrogate females between 4 and 6 months of age underwent separated cohabitation with a vasectomized male at the same time that superovulated females were paired (3 PM) and administered PMSG (see superovulation protocol). The divider was removed 72 hours later at 5 PM. Mating was confirmed visually, and only females who mated received transferred embryos.

Perivitelline injection of lentiviral vector and embryo transfer. High-titer lentiviral vector (~1 × 10^9^ infectious units/ml) was mixed with polybrene for a final concentration of 8 ug/ml, and approximately 100 pl of vector mixture was injected into the perivitelline space using a 1- to 2- um micropipette (as described in Donaldson et al., 2009). Injected embryos were transferred to a single oviduct of psuedopregnant females via the infindibulum. After embryo transfer, surrogate females were placed back in the cage with the vasectommized male partner. Surrogate females were checked for pups starting 18 days after embryo transfer. All pups were born 22-23 days after transfer.

Genotyping by PCR. Genomic DNA was purified from ear-punch tissue using the Gentra Puregene Kit (Quiagen, Valencia, GA). Transgene presence was assayed using forward primer: 5′-caagcagggagctagaacgattc-3′ and reverse primer 5′-caagaacccaaggaacaaagctcc-3′ with the following condiditions: 95 C for 10 min, 30 cycles (95 C for 30s, 55 C for 40s, 72 C for 50s), 72 C for 10 min, and 4 C hold. The resulting product was separated out on a 2% agarose gel, and the presence of a 422-bp fragment indicated amplification of the gfp gene.

## Results

### Experiment 1. Effectiveness of sociosexual manipulation and hormone administration on ovulatory response and embryo production

In studies where it is desired to collect oocytes, for example in IVF, some exposure to a male is necessary; however, for studies where embryos are desired, for example lentiviral mediated transgensis, pre-exposure to a male is not necessary. For oocyte collection, pre-exposure to a male and hormone treatment are necessary for superovulation success (p = 0.03, Fisher’s exact test) (Figure 1a). However, for embryo collection, only hormone administration (p = 0.012, Fisher’s exact test) is necessary for superovulation success (Figure 1b).

**Figure 2:**
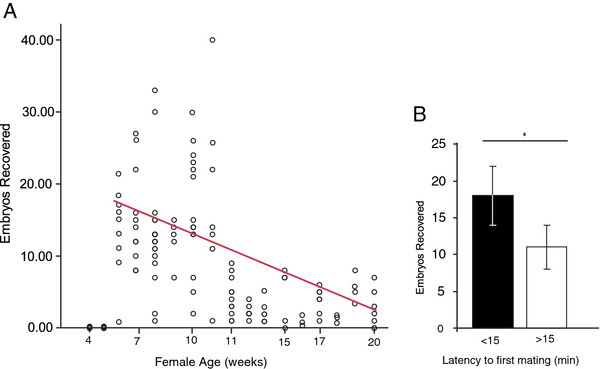
**Female age and occurrence of mating are indicators of superovulation efficiency.** The percentage of female’s not ovulating (black bar), ovulating (gray bar) and superovulating (white bar) following hormone treatment and separated cohabitation in (**A**) the absence of mating and (**B**) the presence of mating. These data suggest that to recover large numbers of oocytes hormone treatment and separated cohabitation are needed for superovulation to occur in the absence of mating. However, for the recovery of embryos hormone treatment and mating alone are sufficient. Treatment group 1.1 (G1.1) did not receive hormone treatment or separated cohabitation. Treatment group 1.2 (G1.2) did receive hormone treatment but did not undergo separated cohabitation. Treatment group 1.3 (G1.3) received hormone treatment and underwent separated cohabitation. Treatment group 2.1 (G2.1) did not receive hormone treatment but did undergo separated cohabitation while Treatment group 2.2 (G2.2) received hormone treatment but did not undergo separated cohabitation. Treatment group 2.3 (G2.3) received hormone treatment and underwent separated cohabitation. Asterisk represents a significant p-value (p < 0.05).

### Experiment 2. Importance of female age and occurrence of mating as indicators of superovulatory success

Regression analysis indicated that female age (p = 0.001, r^2^ = 0.357) is a significant factor contributing to variation in superovulatory response (Figure 2a). Further, the females that mated within 15 minutes of exposure to the male superovulated more than those that did not (*χ*^2^ = 20.82, df = 2, p = 0.00003, Figure 2b).

**Figure 3:**
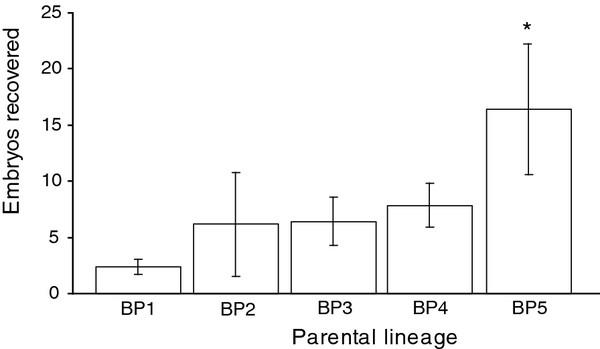
**Impact of parentage on superovulation efficiency.** (**A**) The number of embryos collected per female aged 6-20 weeks significantly decreased with female age. (**B**) Frequency of ovulatory response in females that mated within the first 15 minutes of male access (<15) compared to those that mated after the first 15 minutes of male access. Females that mated within the first 15 minutes of male access were more likely to superovulate than those that did not immediately mate. Error bars are represented as + SEM. Asterisk represents a significant p-value (<0.05).

### Experiment 3. Role of parentage in superovulatory efficiency

Given the variation in ovulatory response seen in prairie voles upon exposure to a male as well as the significant strain variation in mice (see discussion), we were interested in the influence of parentage on superovulatory success in the prairie vole. There was a significant effect of parentage on superovulatory response (F(4,31) = 9.373, p = 0.001, one-way ANOVA). Post hoc analysis revealed that breeder pair 5 responded more efficiently than all other breeder pairs (p = 0.039, Figure 3).

**Figure 4:**
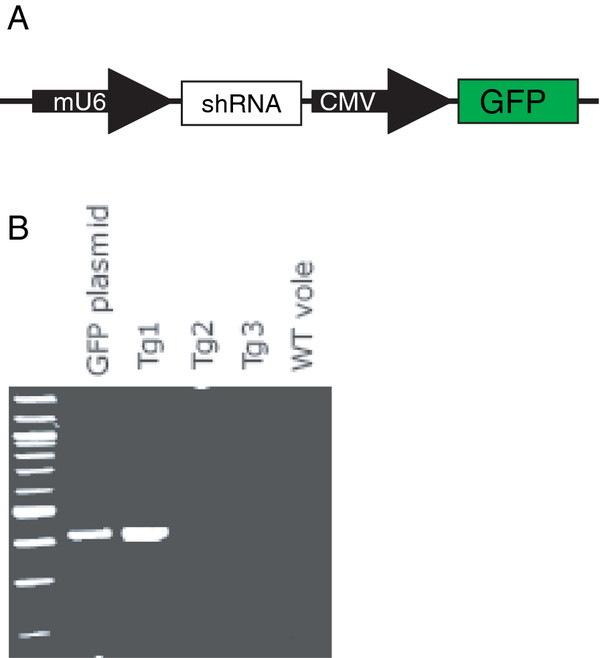
**Generation of transgenic prairie vole. **Mean number of embryos recovered per superovulated female derived from distinct breeder pairs. The numbers of females examined per breeder pair (BP) are as follows: 7 for BP1, 7 for pair BP2, 7 for pair BP3, 9 for pair BP4, and 6 for pair BP5. Posthoc analysis indicates that BP 5 is significantly more sensitive to the superovulation paradigm than the other parental lineages. This data demonstrates that genetic background influences superovulation efficiency in the prairie vole. Error bars are represented as + SEM. Asterisk represents a significant p-value.

### Experiment 4. Generation of transgenic prairie voles

Germline transgenic prairie voles were produced by infecting single-cell embryos with high-titer lentiviral vectors (as described by Donaldson et al., 2009). A total 62 embryos were collected from two experiments from 6 females (10.3 embryos per female). We implanted 30 uninjected embryos and 20 injected embryos into pseudopregnant females. Of these, 4 pups were born from uninjected embryos (13.3%) and 3 from injected embryos (15%). Of the 3 injected embryos, only one individual carried the transgene, as verified by PCR (Figure 4b).

## Discussion

Here we demonstrate that this superovulation method is a viable and effective technique for generating germline transgenic prairie voles. Specifically, we show that pre-exposure to a male in combination with PMSG/hCG is necessary to induce superovulation in the prairie vole for the collection of oocytes (in the absence of mating); however, pre-exposure to a male is not necessary for the collection of embryos in this speices (presence of mating) (Figure 2). The implications of this finding suggest that when fertilized embryos are needed for transgenesis the extra time and resources needed for separated cohabitation are not necessary as reported previously [18]; however, to generate large numbers of oocytes, for example with in vitro fertilization, separated cohabitation is necessary. Thus, some exposure to a male cue is required to achieve ovulation even in the presence of PMSG/hCG. The unusual reproductive physiology of the prairie vole leads to subtle differences that need to be considered when using different types of transgenic technologies.

In addition to social manipulation, other variables known to differ among species, as well as substrains of mice, were examined. The efficiency of superovulation within the prairie vole varies with female age, parentage and latency to first mating bout. Superovulation was most efficient in young females. Notably females aged 6-11 weeks produced a mean of 14 +/- 1.4 embryos while females aged 12-20 weeks produced only a mean of 4 +/- 1.6 embryos. Females that mated within 15 min of access to the male produced significantly more embryos than those that did not. Thus, the latency to mate is a good indicator of oviduction status. Further, there was a significant effect of parentage. For example, 9 out of 9 females from breeder pair 5 superovulated (defined as producing 7 or more embryos), while only 1 out of 6 females superovulated from breeder pair 1. This suggests that some parental lineages of prairie voles are more sensitive to this superovulation paradigm than others. The experiments here suggest that age and genetic background of the female are important factors contributing to superovulatory success, and that occurrence of mating is a good predictor of the number of embryos to be recovered. Further, this work cannot distinguish between the effects of environmental factors as discussed below. This information will dramatically reduce the number of females required, as well as the time and the equipment investment needed to generate a large number of embryos for future gene manipulation studies in this species.

*Why is there variability in ovulatory response?* Previous studies using Illinois derived prairie vole populations have shown that approximately 50% of female prairie voles exposed to males for 2-3 days fail to display lordosis [44] and therefore did not mate or ovulate. In the present study 60% of females exposed to males for 3 days (separated cohabitation only, group 2.1) ovulated. When females underwent the superovulation paradigm 80% of the females showed an ovulatory responses, with 52.5% of those females superovulating (group 2.3). This suggests that the superovulation protocol decreases the variability previously seen in mating and ovulatory response; however, some of the variation remains unaccounted for. Such variation could be explained by environmental and/or genetic differences among individual females.

It is possible that the social environment in the natal nest (i.e. parental nurturing behavior, juvenile-juvenile interactions) could be factors that result in long-lasting neurochemical changes that increase reproductive potential as adults. For example females are more likely to engage in affiliative behavior as adults if they remain in the natal nest after weaning [45]. The presence of the father in the natal nest also increases their propensity to form a partner preference as adults [46,47]. These studies suggest that the social environment during development, perhaps when the pups are interacting with their parents and siblings, is critical for shaping factors that result in long-lasting neurochemical changes that impact reproductive potential as adults.

In addition to environmental factors, genetic background could also be playing a role in the variation seen in ovulatory response among female prairie voles. In laboratory *Mus* species, the average number of embryos induced by superovulation is highly strain-dependent. Females of the strains B6, BALB/cByJ, 129/SvJ, CBA/CaJ, SJL/J, C58/J are considered high responders to superovulation and can be induced to ovulate 40-60 embryos while females of the strains A/J, C3H/HeJ, BALB/cJ, 129/J 129/ReJ, DBA/2 J, C57L/J are considered low responders and produce at most 15 embryos per mouse [28]. This suggests that subtle differences in genotype can have dramatic consequences on the expression of this particular reproductive trait. Studies in female prairie voles have shown that there is a graded ovulatory response to prolonged social stimulation and that some females show exceptional ovulatory sensitivity following mating with only 6-12 hours of male exposure [38]. In the present study 100% of females from breeder pair 5 superovulated while only 16% of females from breeder pair 1 superovulated, demonstrating that some lineages of prairie voles are more sensitive to this superovulation paradigm than others. Prairie voles are maintained as an outbreed population; however, as transgenesis in this species matures it may become necessary to establish optimal breeding lines for superovulation and embryo production as has been done in the mouse.

## Conclusion

In conclusion, the present study has identified a superovulation and mating paradigm that allows for the recovery of a large number of developmentally synchronized oocytes and embryos that can be used for the production of transgenic prairie voles. However, this paradigm is not perfect and variation in ovulatory response still exists. Several variables warrant further studies. First, the diet provided to a prairie vole breeding colony may have important consequences on the reproductive responsiveness of offspring. Second, increasing the hours of light per day (for example from 14:10 to 18:6) may increase the ovulatory response of hormone treated females. And, finally it may prove necessary to establish optimal donor and recipient strains as prairie vole transgenesis matures.

## Competing interests

The authors declare that they have no competing interests.

## Authors’ contributions

AK, MM and LY contributed to conception and design. AK, MM and CB contributed to acquisition of data. CJ contributed to embryo transfer technique. AK and LY contributed to analysis and interpretation of data. AK wrote the manuscript and MM, CB and LY contributed to editing the manuscript. All authors read and approved the final manuscript.
